# Effect of psyllium husk on low anterior resection syndrome after rectal cancer surgery—a pilot prospective cohort study

**DOI:** 10.3389/fsurg.2025.1686486

**Published:** 2025-12-09

**Authors:** Silje Stensholt Holte, Natalia Avaliani, Geir Hoff, Airazat M. Kazaryan, Keson Jaioun, Giedrius Lauzikas, Johannes Kurt Schultz

**Affiliations:** 1 Department of Gastrointestinal Surgery, Telemark Hospital Trust, Skien, Norway; 2 Faculty of Medicine, Institute of Clinical Medicine, University of Oslo, Oslo, Norway; 3 Department of Research and Innovation, Telemark Hospital Trust, Skien, Norway; 4 Faculty of Medicine, European University, Tbilisi, Georgia; 5 Department of Gastrointestinal Surgery, Østfold Hospital Trust, Grålum, Norway; 6 Department of Surgery N1, Yerevan State Medical University After M. Heratsi, Yerevan, Armenia; 7 Department of Surgery N2, I.M.Sechenov First Moscow State Medical University, Moscow, Russia; 8 Department of Gastrointestinal Surgery, Akershus University Hospital, Lørenskog, Norway

**Keywords:** bowel dysfunction, LARS, low anterior resection, rectal resection, rectal cancer, quality of life, psyllium husk

## Abstract

**Introduction:**

Low anterior resection syndrome (LARS) is a frequent, undesired consequence of rectal cancer surgery. Psyllium husk has been suggested as a nutritional supplement in the management of LARS, but without trial-based evidence of its effect. In preparation of a randomized clinical trial, this pilot study aimed to estimate the effect of Psyllium husk in patients with LARS, determining the treatment duration required to observe the effect and assess patient compliance.

**Methods:**

This single arm phase II study included patients with LARS score >20 ten months or more after rectal resection. Patients who consented to participate were given 3.66 g Psyllium husk fiber twice daily for 56 days. The primary endpoint was severity of bowel dysfunction using the LARS score. The secondary endpoint was quality of life (QoL) using EQ-VAS score in the EQ-5D-5L questionnaire. A reduction of 7 points in LARS score was considered clinically relevant. Questionnaires and scores were collected on paper day 0 (baseline), day 28 and day 56.

**Results:**

22 patients were assigned to the intervention and included in the analysis. The average age was 65.3 (SD 10.4), the average LARS score at baseline was 35,7 (SD 3.5), four patients had received radiotherapy. The proportion of patients with a LARS score reduction of 7 points or more compared to baseline was 50.0% at day 28 (11/22) and 50.0% at day 56 (10/20). The mean reduction in LARS scores from baseline to 8 weeks of treatment was 7.7 points. At day 28, the proportion of patients with a QoL score (EQ-VAS score) increase of 10 points or more was 36.4% (8/22), increasing to 60.0% (12/20) by day 56. Two patients dropped out after 4 weeks of treatment, one due to taste and consistency of the supplement and one due to inconsistently beneficial effect.

**Conclusions:**

Psyllium husk appears to clinically significantly improve both bowel dysfunction and QoL in rectal cancer patients suffering from LARS. This study highlights the need for further systematic research, and provides a strong basis for a well-designed RCT.

## Introduction

Advancements in chemotherapy, radiotherapy, and surgical techniques for rectal cancer have led to significant improvement in 5-year survival rate from 15% in 1975 (1) to 73% in 2020 (89% for stage I-III cancer) in Norway (2). Quality of life (QoL) after treatment has therefore become increasingly important in long-term patient care. The dominant surgical procedure for rectal cancers is a sphincter preserving low anterior resection (LAR) with creation of a colorectal or coloanal anastomosis. This often leads to a long-term bowel dysfunction, commonly referred to as low anterior resection syndrome (LARS) (3). LARS is characterized by a set of symptoms including fecal incontinence, frequent and/or urgent bowel movements, loose stools and incomplete defecation. The LARS score questionnaire was developed to capture the severity of the syndrome. It ranges from 0 to 42 and is divided into three categories—no LARS (0–20), minor LARS (21–29) and major LARS (30–42) (4). A recent international meta-analysis estimated the prevalence of major LARS after sphincter-preserving surgery to be around 40% (5). In the Norwegian LaTE study, up to 75% of patients treated with neoadjuvant radiotherapy experienced major LARS at a median of 7.4 years after surgery (6). In Scandinavia, 79% of patients report LARS symptoms after LAR, with over 50% experiencing major LARS impacting their QoL (7, 8). Major LARS often impairs both QoL and daily functioning, and in many cases, leads to social withdrawal and inability to work.

Various interventions have been suggested to alleviate the symptoms of LARS, but very few randomized trials have been carried out to reliably evaluate their effectiveness (9, 10). Up to date, mainly small observational studies exist for many possible interventions (10–12). Sacral nerve stimulation and trans-anal irrigation have shown promising results (10, 13, 14), whereas the effectiveness of other interventions is poorly investigated. Conservative symptom-reducing measures, such as dietary management, laxatives, bulking agents, anti-diarrheal or spasmolytic agents are commonly applied, but no specific treatment is systematically recommended due to lack of robust evidence (1, 11, 12).

Psyllium husk is a soluble, non-fermentable fiber from the genus Plantago seed husk which forms a gel in the intestines when hydrated, helping to normalize stool consistency in conditions like constipation, diarrhea, and IBS (15). While Psyllium has shown effectiveness in treating fecal incontinence in women (16), obstructed defecation after Stapled Trans Anal Resection of the Rectum (STARR) (17) and constipation (18), no clinical studies have specifically evaluated its effect in patients with LARS (19), despite promising case reports (20). As Psyllium husk changes stool consistency, it may alter both incontinence for stool, frequently associated with the incontinence-dominant LARS pattern (21), and frequency and fragmentation associated with the frequency-dominant pattern.

The primary aim of this phase II study was to estimate the effect of Psyllium husk in rectal cancer patients suffering from LARS, and the required time until clinical effect occurs. Secondary aims were to estimate patient coherence to the intervention and the relation between LARS score and QoL score in preparation for a randomized clinical trial.

## Methods

### Study design

We designed a prospective, non-comparative feasibility cohort study. All participants were recruited at Telemark Hospital Trust. The study was approved by the Regional Ethics Committee, Southeast Norway (reference number 732141), as well as the local data protection officer at Telemark Hospital Trust (reference number 23–21). The study was conducted in accordance with the ethical principles stated in the declaration of Helsinki and guidelines on GCP. The study was registered with ClinicalTrials.gov (NCT06724198). STROBE guidelines were followed for reporting this study (22).

### Eligibility criteria and inclusion

Eligibility criteria are summarized in Table 1. Patients over 18 years of age operated after January 01, 2021 with low anterior resection for rectal cancer (stage I-IV) at Telemark Hospital Trust were assessed for eligibility in the local quality register or at routine follow-up. Patients reporting a LARS score >20 (minor and major LARS) at least 10 months or more after primary surgery and at least two months after stoma reversal were eligible. Patients unable to sign written consent or unable to understand and answer the questionnaires were excluded, as were patients with any contraindication to the intervention (upper GI obstruction, inherited fructose intolerance, glucose-galactose malabsorption and sucrase-isomaltase deficiency). All 22 participants provided written informed consent. Patients using daily bowel regulatory medication prior to inclusion were advised to continue the same treatment throughout the study. Eligible patients were either approached at routine follow-up visits or contacted by telephone. Two patients were recruited through an announcement in social media. One surgeon (SSH) informed and included all patients in the trial.

**Table 1:** Inclusion and exclusion criteria.

Inclusion Criteria	Exclusion criteria
Operated with low rectal resection for rectal cancer	Contraindications to Psyllium husk:
	- hypersensitivity - intestinal obstruction or reduced esophageal function - rare congenital medical conditions such as sucrase-isomaltase deficiency, fructose intolerance and glucose-galactose malabsorption
LARS score >20 at 12 months or more after surgery	LARS score < 21
Written consent	Various conditions rendering the patient unable to answer questionnaire

### Interventions

3.66 g Psyllium husk was given orally twice daily for 8 weeks. All patients received full supply of the supplement at inclusion. They recorded in a daily diary when the Psyllium supplement was taken and returned any remaining supplement to check for compliance.

### Endpoints

The primary endpoint was the difference in LARS score between baseline and after 56 days of treatment with Psyllium husk. Secondary endpoints were changes in LARS score after 28 days, and changes in QoL (EQ-5D-5L EQ-VAS score) at both time points. EQ-5D-5L is a health status measure consisting of 5 multiple-choice questions covering 5 health dimensions with 5 alternative answers for each. In addition, it contains the EQ-VAS, a visual analog scale for overall QoL ranging from 0 to 100 used to assess health-related QoL with increased scores indicating improved QoL.

### Data collection and follow-up

Patients baseline characteristics (age, gender, BMI, ASA score, perioperative radio chemotherapy, type of surgery, anastomotic level and technique, any anastomotic complication and previous diverting ileostomy) were collected from the local prospective quality registry and hospital electronic medical records (EMR).

LARS severity was assessed using the validated Norwegian LARS score questionnaire (4, 23–25) and QoL was assessed using the EQ-5D-5L questionnaire (26, 27). Patients were contacted by telephone after the initial screening; LARS scores and subjective complaints were collected during the first telephone contact for eligibility. Informed consent form, questionnaires and diary were sent by post along with instructions on how and when to fill out the questionnaires. Written informed consent and baseline scores was obtained prior to the start of the intervention. Questionnaires were returned by post after 4 and 8 weeks of treatment in separate envelopes. Safety was assessed by monitoring adverse effects during the entire study period through regular follow-ups by telephone. Adverse effects were classified as mild or moderate, based on the low side-effect profile of the investigational substance (Psyllium husk).

After finishing the study, all patients were offered the use of the supplement by prescription beyond the study period with a planned 6 months follow-up by phone to evaluate the number of patients still using the supplement and its long term effect. Data was collected in Ledidi Core®, an electronic platform for secure data collection, storage and analysis.

### Statistical analysis

This feasibility study was designed as a uncontrolled single-cohort study with the patients being their own controls. A reduction in LARS score ≥7 points was considered clinically relevant, consistent with previous studies using LARS scores as an endpoint (9). Similarly, improvement of ≥10 points in the EQ-VAS score has been considered clinically significant (28). Additionally, we defined the responders as patients who have clinically meaningful improvement in LARS score of ≥7 points from baseline to 56 days of treatment.

The main hypothesis was that patients using Psyllium husk would have a clinically significant decrease in mean LARS score over time. To detect a 7-point difference with 80% power and a two-sided significance level of 5%, a minimum of 9 paired observations was required. Accounting for an anticipated dropout rate of 20% dropout rate, the adjusted sample size was calculated to be 12 pairs. In addition to maintaining statistical robustness, we planned a larger sample size and set it to *n* = 22 to allow subgroup analysis and enhance generalizability across a broader population. It also provides buffering against protocol deviations and variability in adherence, which could detect data quality. Analyses were based on the per-protocol population.

Descriptive statistics were used to summarize the study population. Continuous variables were reported as mean, standard deviation (SD) or median and interquartile (IQR) when appropriate. Categorical variables were expressed as frequencies and relative percentages. Paired t-test and Wilcoxon signed-rank test were used to investigate the changes in LARS score and QoL score over time. Additionally, independent t-test were also performed to investigate the difference between responders and non-responders, and Chi-square test or Fisher's exact test were used to investigate relationship between two independent categorical variables. McNemar's test was used to compare proportion of patients reporting problems between baseline and after 8 weeks of treatment in the EQ-5D QoL domains regarding (1) mobility, (2) self-care, (3) usual activities, (4) pain/discomfort and (5) anxiety/depression.

All statistical analyses were performed using STATA version 18. (StataCorp, College Station, TX, USA) and RStudio version 2025.05.0 + 496 Statistical Software. All *p*-values were 2-sided and *p*-value <0.05 were considered statistically significant.

## Results

### Study population and protocol compliance

**Figure 1:**
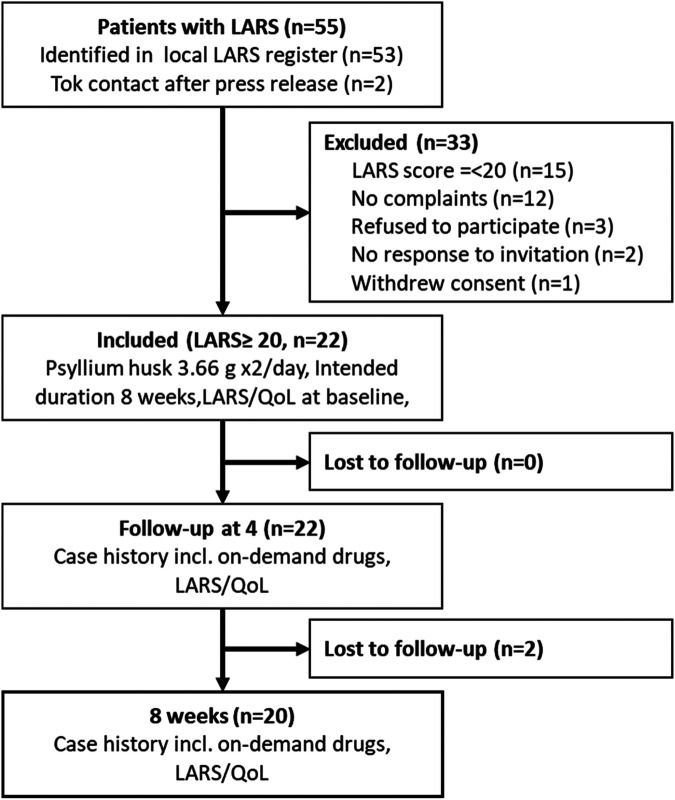
Flow diagram of the study. Out of 55 screened patients, 22 were assigned to the study intervention and included in the analysis. Two patients dropped out after 4 weeks of treatment. *LARS, low anterior resection syndrome; QoL, quality of life.

Patients were recruited between January 30 and March 26, 2025. The flow diagram of the study is presented in Figure 1. Of 53 patients screened in the internal quality register from July 01, 2021, 15 patients had a LARS score <21 and by definition no LARS. 38 patients had a LARS score >20. Twelve patients with LARS score >20 were not bothered by their bowel dysfunction and were therefore not interested in the intervention, three refused to participate due to other health issues, one withdrew consent, and two didn't answer. Two eligible patients took contact after press release. Thus, 22 patients were assigned to the intervention.

The baseline characteristics of the patients included in the analysis are summarized in Table 2.

**Table 2:** Demographics and baseline characteristics.

Characteristics	LARS score > 20 *n* = 22
Age, mean: (SD)	65.3 (10.4)
Sex: male (%)	11 (50.0)
BMI, mean *n* (SD)	25.1 (3.2)
ASA score, mean (SD)	2.1 (0.60)
Prior radiotherapy*, *n* (%)	4 (18.2)
Prior ileostomy, *n* (%)	10 (45.5)
Date FA—study start, mean days (SD)	580 (236)
Days with temporary stoma, mean (SD)	124 (100)
Prior adjuvant chemotherapy, *n* (%)	4 (18.2)
Side-to-End Anastomosis, *n* (%)	17 (77.3)
End-to-End Anastomosis, *n* (%)	5 (22.7)
Level of anastomosis from AV, mean cm (SD)	5.9 (2.2)
Anastomotic leakage, *n* (%)	3 (13.0)
Pathology T-stage ≥ 3, *n* (%)	12 (54.5)
Pathology N-stage ≥ 1, *n* (%)	5 (22.7)
Time from primary surgery to inclusion, mean days (SD)	750 (344)
Baseline LARS score, mean (SD)	35.7 (3.5)
Baseline EQ-VAS score**, mean (SD)	66.9 (16.5)
LARS categories at baseline major vs. minor	21 vs. 1

FA, functional anastomosis; AV, indicates anal verge.

* Prior neoadjuvant radiochemotherapy *n* = 2.

** EQ-VAS score 0–100.

Two patients interrupted the treatment after the first 4 weeks of treatment, one due to difficulties taking the supplement orally (taste, consistency) and one due inconsistently beneficial effect over the first 4 weeks. One patient reduced the treatment dose to once daily after 4 weeks of treatment due to constipation. The remaining 19 patients completed the 8 weeks treatment with dosage twice a day. During the study, we recorded no serious adverse effects.

### LARS score

**Figure 2:**
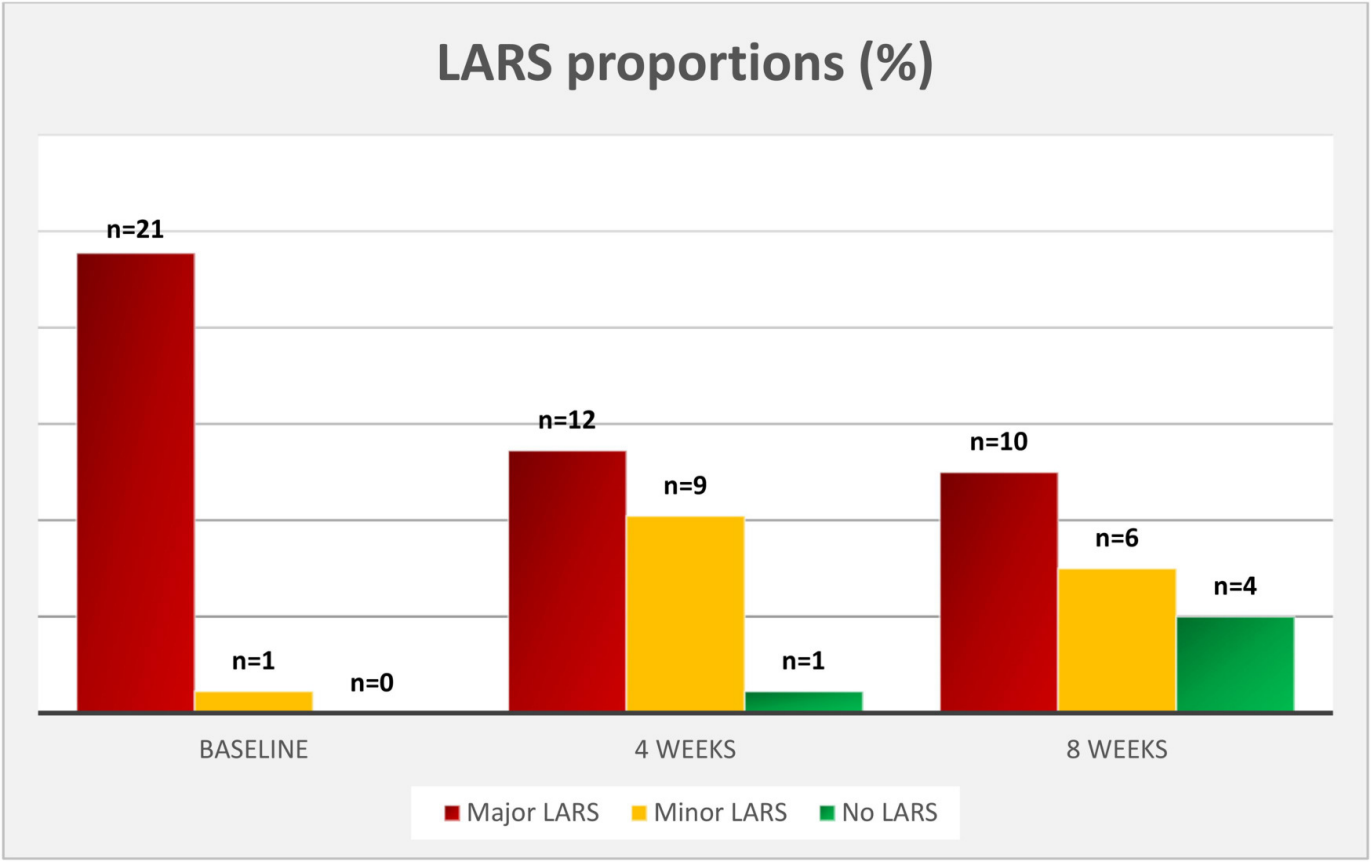
Changes in major, minor and no LARS* over the 8-week treatment period. Proportion of patients with major LARS decreased from 95.5% at baseline to 50.0% after 8 weeks of treatment. 20% of the patients decreased in LARS score to category “no LARS”. *LARS indicates low anterior resection syndrome.

**Figure 3:**
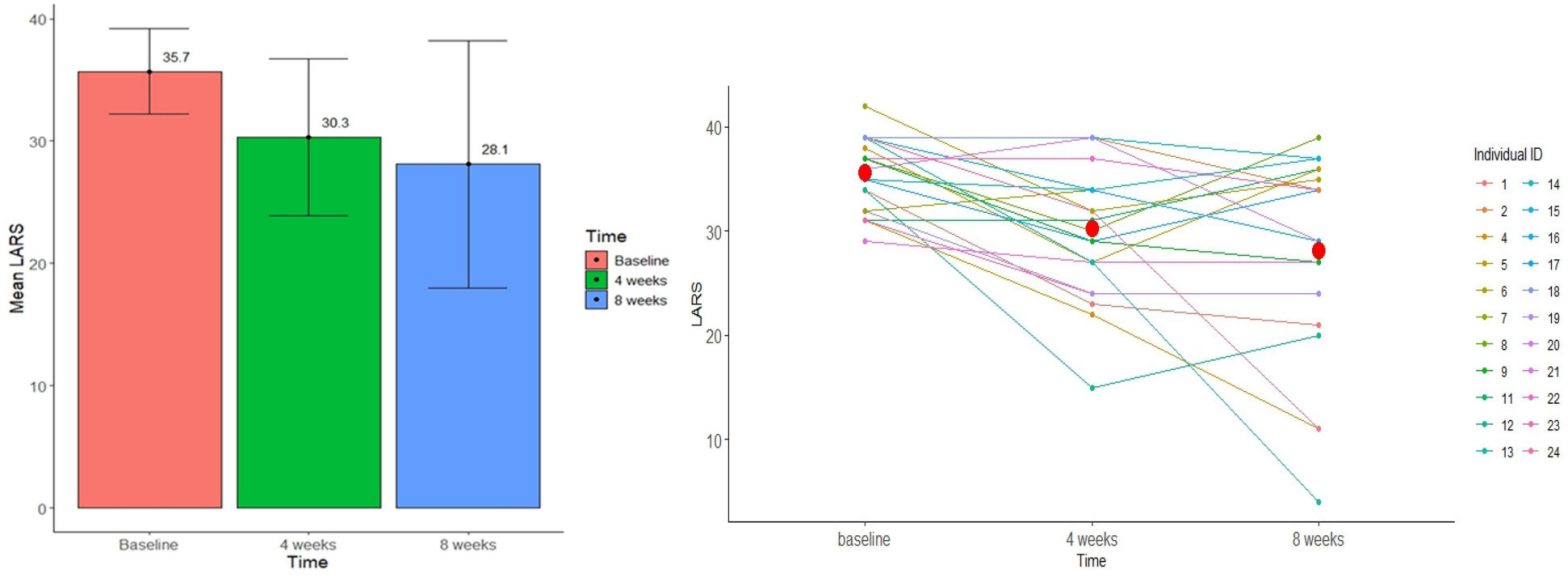
Changes in LARS score during the 8-week study period showing mean decrease and LARS score and its variability.

Twenty-two patients completed the LARS score at baseline and after 4 weeks of treatment, and 20 of those completed LARS score after 8 weeks of treatment. The changes in LARS score are presented in Table 3. The mean LARS score decreased significantly from 35.7 (SD = 3.5) at baseline to 30.3 (*n* = 22, SD = 6.4, *p* = <0.001) after 4 weeks and to 28.1 (*n* = 20, SD = 10.1, *p* = 0.004) after 8 weeks of treatment. The mean difference in LARS score between baseline and after 8 weeks of treatment was 7.7 [*n* = 20, 95% CI: (2.8, 12.5), *p* = 0.004]. There was no significant difference in LARS scores from 4 to 8 weeks treatment (*p* = 0.294). The distribution of major, minor and no LARS at the different time points is presented in Figure 2. The proportion of patients with major LARS decreased from 21/22 (95.5%) to 12/22 (54.5%) after 4 weeks and to 10/20 (50.0%) after 8 weeks of treatment. Changes in LARS score during the 8-week study period are shown in Figure 3.

**Table 3:** Changes in LARS score and QoL score over time.

Scales	Baseline (*n* = 22)	After 4 weeks (*n* = 22)	After 8 weeks (*n* = 20)	Mean difference^a^ (SD); 95% CI	Mean difference^b^ (SD); 95% CI	Mean difference^c^ (SD); 95% CI	*p*-value^a^ 0/4 weeks	*p*-value^b^ 0/8 weeks	*p*-value^c^ 4/8 weeks
LARS									
Major LARS^d^, *n* (%)	21 (95.5)	12 (54.5)	10 (50.0)						
LARS score reduction min. 7 points^e^, n		11	10						
LARS score reduction >20%, *n*		9	9						
LARS score, median (IQR)	36.5 (32–39)	30.5 (27–34)	31.5 (22.5–36)				<0.001	0.002	0.613
LARS score, mean (SD)	35.7 (3.5)	30.3 (6.4)	28.1 (10.1)	5.4 (5.6); (2.9, 7.8)	7.7 (10.4); (2.8, 12.5)	2.1 (8.8); (−2.0, 6.2)	<0.001	0.004	0.294
Quality of life									
QoL score increase^f^, n		8	12						
QoL score, mean (SD)	66.9 (16.5)	73.9 (11.9)	77.8 (12.0)	6.9 (8.3); (3.2, 10.6)	12.1 (12.9); (6.0, 18.2)	4.8 (7.5); (1.2. 8.3)	<0.001	<0.001	0.011
QoL score, median (IQR)	70 (55–75)	75 (65–80)	80 (70–85)				<0.001	<0.001	0.004

a LARS from baseline to 4 weeks treatment, *n* = 22.

b LARS from baseline to 8 weeks treatment, *n* = 20.

c LARS from 4 to 8 weeks treatment, *n* = 20.

d Major LARS score: 30–42.

e LARS score reduction indicates a reduction of 7 points or more.

f QoL score increase indicates an increased QoL score of 10 points or more.

QoL score indicates EQ-VAS score 0–100.

### Quality of life

**Figure 4:**
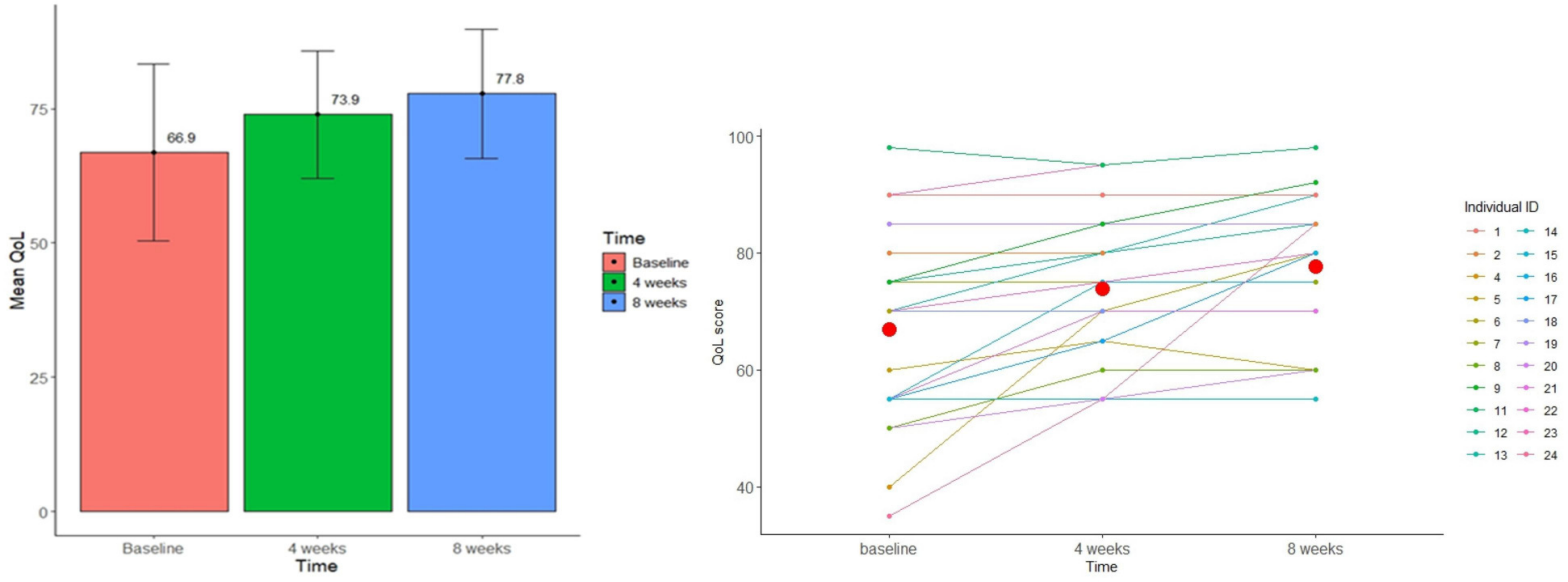
Changes in EQ-VAS score during the 8-week study period showing the mean increase and variability in EQ-VAS score.

Twenty-two patients completed the EQ-5D-5L questionnaire at baseline and at 4 weeks and 20 patients completed EQ-5D-5L at baseline, at 4 weeks and at 8 weeks. After the first 4 weeks of treatment, the proportion of patients with an increased QoL EQ-VAS of 10 points or more was 36.4% (8/22). After 8 weeks of treatment the proportion was further increased to 60.0% (12/20). Mean (SD) EQ-VAS score increased from 66.9 (16.5) at baseline to 73.9 (11.9) after 4 weeks of treatment. After 8 weeks of treatment, mean (SD) EQ-VAS further increased to 77.8 (12.0). The mean difference in EQ-VAS score from baseline to 8 weeks was 12.1 [*n* = 20, 95% CI: (6.0, 18.2), *p* = <0.001]. The mean changes in QoL EQ-VAS scores are summarized in Table 3 and presented in Figure 4. The changes in the different EQ-5D-5L dimensions are presented in Table 4. The proportion of patients reporting pain/discomfort and anxiety/depression was decreased after 8 weeks of treatment, while the other 3 dimensions remain unchanged.

**Table 4:** Changes in EQ-5D domain over 8 weeks treatment.

Level of problems	EQ-5D-5L dimensions									
	Mobility		Self-care		Usual activities		Pain/discomfort		Anxiety/depression	
	Baseline	8 weeks	Baseline	8 weeks	Baseline	8 weeks	Baseline	8 weeks	Baseline	8 weeks
	*n* = 22	*n* = 20	*n* = 22	*n* = 20	*n* = 22	*n* = 20	*n* = 22	*n* = 20	*n* = 22	*n* = 20
0 No problems, *n* (%)	16 (72.7)	14 (70.0)	20 (90.9)	19 (95.0)	15 (68.2)	14 (70.0)	5 (22.7)	10 (50.0)	12 (54.6)	15 (75.0)
1 Slight problems, *n* (%)	5 (22.7)	5 (25.0)	2 (9.1)	1 (5.0)	3 (13.6)	5 (25.0)	12 (54.6)	7 (35.0)	7 (31.8)	3 (15.0)
2 Moderate problem, *n* (%)	1 (4.5)	1 (5.0)	0 (0)	0 (0)	3 (13.6)	1 (5.0)	5 (22.7)	3 (15.0)	3 (13.6)	2 (10.0)
3 Severe problems, *n* (%)	0 (0)	0 (0)	0 (0)	0 (0)	1 (4.6)	0 (0)	0 (0)	0 (0)	0 (0)	0 (0)
4 Unable/extreme, *n* (%)	0 (0)	0 (0)	0 (0)	0 (0)	0 (0)	0 (0)	0 (0)	0 (0)	0 (0)	0 (0)
5 Proportion reporting any problems, *n* (%)	6 (27.3)	6 (30.0)	2 (9.1)	1 (5.0)	7 (31.8)	6 (30.0)	17 (77.3)	10 (50.0)	10 (45.5)	5 (25.0)
*p*-value*	>0.999		>0.999		>0.999		0.031		0.125	

* *p*-value from the McNemar test to investigate differences in proportions of patients reporting any problem. The proportion of patients reporting pain/discomfort and anxiety/depression decreased after 8 weeks of treatment, while the other problems remained unchanged during the study period.

### Characteristics of patients not responding to treatment

Eighteen of 22 patients improved in LARS scores during the intervention period, while four (all females) experienced increased LARS scores. Responders (*n* = 10) were defined as patients who achieved a clinically meaningful reduction in LARS score of ≥7 points following 8 weeks of treatment. The characteristics of non-responders (*n* = 10) vs. responders are summarized in Table 5. Non-responders were more likely to be female and had a higher mean anastomosis level (6.6 cm, SD 2.3) compared to responders (5.6 cm, SD 4.0). At baseline, non-responders had a slightly lower mean LARS score compared to responders (35.2, SD 3.5 vs. 36.3, SD 3.5). After 8 weeks of treatment, responders demonstrated a higher QoL score (mean 81.2, SD 10.2) compared to non-responders (mean 74.3, SD 13.2). Nevertheless, non-responders showed a notable improvement in a mean QoL score of 9 points from baseline (mean 65.3, SD 15.2) to 8 weeks of treatment (mean 74.3, SD 13.2).

**Table 5:** Characteristics of non-responders vs. responders.*

Characteristics	Non-responders (*n* = 10)	Responders (*n* = 10)	*p*-value
Major LARS at baseline, n	9 (90.0)	10 (100)	
Age, mean (SD)	66.6 (10.5)	64.7 (11.0)	0.698
Gender; female, *n* (%)	7 (70.0)	3 (30.0)	0.074
ASA score, mean (SD)	2.2 (0.6)	2.0 (0.7)	0.500
BMI, mean (SD)	25.3 (3.4)	25.1 (3.0)	0.868
QoL baseline, mean (SD)	65.3 (15.2)	66.0 (18.4)	0.927
QoL 8 weeks, mean (SD)	74.3 (13.2)	81.2 (10.2)	0.207
LARS baseline, mean (SD)	35.2 (3.5)	36.3 (3.5)	0.492
LARS 8 weeks, mean (SD)	35.1 (3.3)	21.1 (9.8)	<0.001
Anastomosis cm, mean (SD)	6.6 (2.3)	5.6 (4.0)	0.311
Previous diverting stoma, %	3 (30.0)	5 (50.0)	0.650

* Responders are defined as patients with a LARS score reduction of >7 points.

## Discussion

In this phase II cohort study, the daily use of Psyllium husk, improved both bowel function and QoL in a large proportion of rectal cancer patients suffering from LARS. LARS is one of the main challenges in rectal cancer survivors with major impact on QoL. Psyllium husk, which is currently used to treat fecal incontinence and IBS, is an affordable and well tolerated supplement. The effect of Psyllium husk on LARS observed in this study strengthens the hypothesis that its use may be truly beneficial with the potential to improve QoL for rectal cancer survivors considerably. The difference in mean LARS score of 7.7 would bring many patients from major to minor or from minor to no LARS and is therefore considered clinically relevant. Importantly, no serious adverse events were reported. Only minor tolerability issues were observed: one patient discontinued treatment due to difficulties with the taste and consistency, and another reduced the dose because of constipation. These events highlight however the importance of monitoring individual responses to the supplement and tailoring the treatment.

Compared to the effect of other conservative measures for LARS, Psyllium husk appears promising.

Dietary adjustments are commonly recommended for this patient group, but lack standardized protocols and robust evidence (1). Although loperamide is frequently used for urgency and incontinence among LARS patients, its effect on overall LARS symptoms is limited due to the complexity of the condition, while causing more often constipation compared to Psyllium husk in fecal incontinence (20). Probiotics have shown potential in modulating intestinal microbiota, but clinical studies in LARS-specific populations have not yet demonstrated any significant bowel improvement related to its use (10). However, some reports suggest that frequency-dominant LARS patients have decreased diversity of gut microbiome with lower levels of lactic acid-producing bacteria (29). Transanal irrigation has demonstrated efficacy in randomized trials, particularly for persistent and severe LARS (13), but requires training, equipment, and patient motivation, which may limit its accessibility. In contrast, Psyllium husk is inexpensive, widely available, easy to administer, and tolerated well by most patients, making it a feasible first-line option in clinical practice. However, due to the complexity of the condition, variability of symptoms and individual patient factors, personalized treatment approaches are essential to optimize individual outcomes.

One may discuss whether the observed difference in LARS is clinically relevant. To our knowledge, minimal importance difference (MID) for LARS scores has not yet been investigated properly, but a minimum of 5-point difference in LARS score has been suggested as a minimal clinically important difference (MCID) (30). A reduction of 7 points in LARS score is considered clinically significant in practice, as it often reflects a shift from major LARS to minor LARS, as demonstrated in this study. While this threshold is not formally validated as a clinically significant change in LARS score, this level of change often represents a noticeable improvement in symptoms and aligns with previous clinical studies using LARS score as outcome measures (9, 31).

We assessed QoL by using the EQ-5D-5L questionnaire, considering an increase of 10 points or more in the EQ-VAS score as clinically significant. The 10 point increase is based on the minimal important difference (MID) for EQ-VAS score in cancer patients, which typically ranges from 7 to 10 points according to the literature (28). Previous studies also support a 10-point increase as a meaningful change, reflecting an improvement that patients perceive as significant in their overall QoL (32). The EQ-5D-5L questionnaire is brief with an visual analog scale (EQ-VAS) used as a quantitative measure of health outcome that reflects the patient's own judgement, which is useful for capturing subjective changes in the patient outcomes (27). In our study, we found that 70.0% of patients reported an improvement in QoL after 8 weeks of treatment, while 30% experienced no change. Notably, no patients reported a decline in QoL, even though four individuals had increased LARS scores after 8 weeks. In the separate domains in EQ-5D-5L questionnaire, we found significant decrease in proportion of patients reporting pain/discomfort and anxiety/depression after 8 weeks of treatment compared to baseline (Table 4). The broad domains in the EQ-5D-5L may reduce its sensitivity to specific LARS-related symptoms, such as bowel dysfunction, and therefor may fail to demonstrate significant improvements in this patient population. To enhance the assessment of treatment effects in future trials, more condition-specific questionnaires such as EORTC QLQ-C30 and CR29 should be considered.

All our patients were operated with minimal invasive surgery (laparoscopic or robotic) with a minimum of 10 months bowel restitution (and anticipated spontaneous relief of symptoms) before the start of the intervention. In this study, we observed a high percentage of patients with prior ileostomy (45.5%) and prior radiotherapy (18.2%), both known to influence bowel function and contribute to the severity of LARS symptoms. We examined these variables to explore potential confounding factors and to identify subgroups that might benefit differently from Psyllium husk treatment, but the small sample size limited subgroup analysis.

As a pilot study designed to inform a future RCT, this study has some limitations. The simplified design and small sample size reflects its feasibility focus. Recruitment, follow-up, and outcome assessment were performed by a single surgeon (SSH), who also treated many of the included patients. This may introduce selection bias, as patients with established clinical relationships may be more likely to consent to participation, potentially skewing the sample toward more motivated or compliant participants. Additionally, observer bias may influence the outcome assessment, particularly in QoL measures, where patients may report improved scores due to perceived expectations with the investigator. While standardized questionnaires were used to minimize subjective interpretation, the lack of blinding and independent assessment remains a limitation. These factors state the importance of implementing blinded outcome assessment and independent recruitment in future multicenter randomized trials to reduce bias and enhance generalizability. Given the feasibility design of the study, concurrent use of other medications, supplements or measures may have had a confounding effect on the results. However, since patients had persistent bowel dysfunction unresponsive to other measures, the improvement observed after adding Psyllium husk suggests a genuine effect of the supplement.

The findings of this study underline the need for a more thorough and systematic investigation of the effect of Psyllium husk on LARS score. The study provides an important and solid foundation for planning a well-designed randomized controlled trial. As a result of this study, we are in the process of finalizing the protocol for a double-blind crossover multicenter randomized trial that will be followed by a dose response trial. This will give us the possibility to study the effect of Psyllium husk in different subgroups of patients with LARS. The protocol for this upcoming trial will shortly be available in the Clinical Trials Information System (CTIS).

## Data Availability

The raw data supporting the conclusions of this article will be made available by the authors, without undue reservation.
