# Supplementary material for: Effect of psyllium husk on low anterior resection syndrome after rectal cancer surgery—a pilot prospective cohort study
Source: Front Surg. 2025 Dec 9;12:1686486. doi: 10.3389/fsurg.2025.1686486 (PMC12722911; doi:10.3389/fsurg.2025.1686486)
Supplement: Supplementary file 1 [file Image1.pdf]

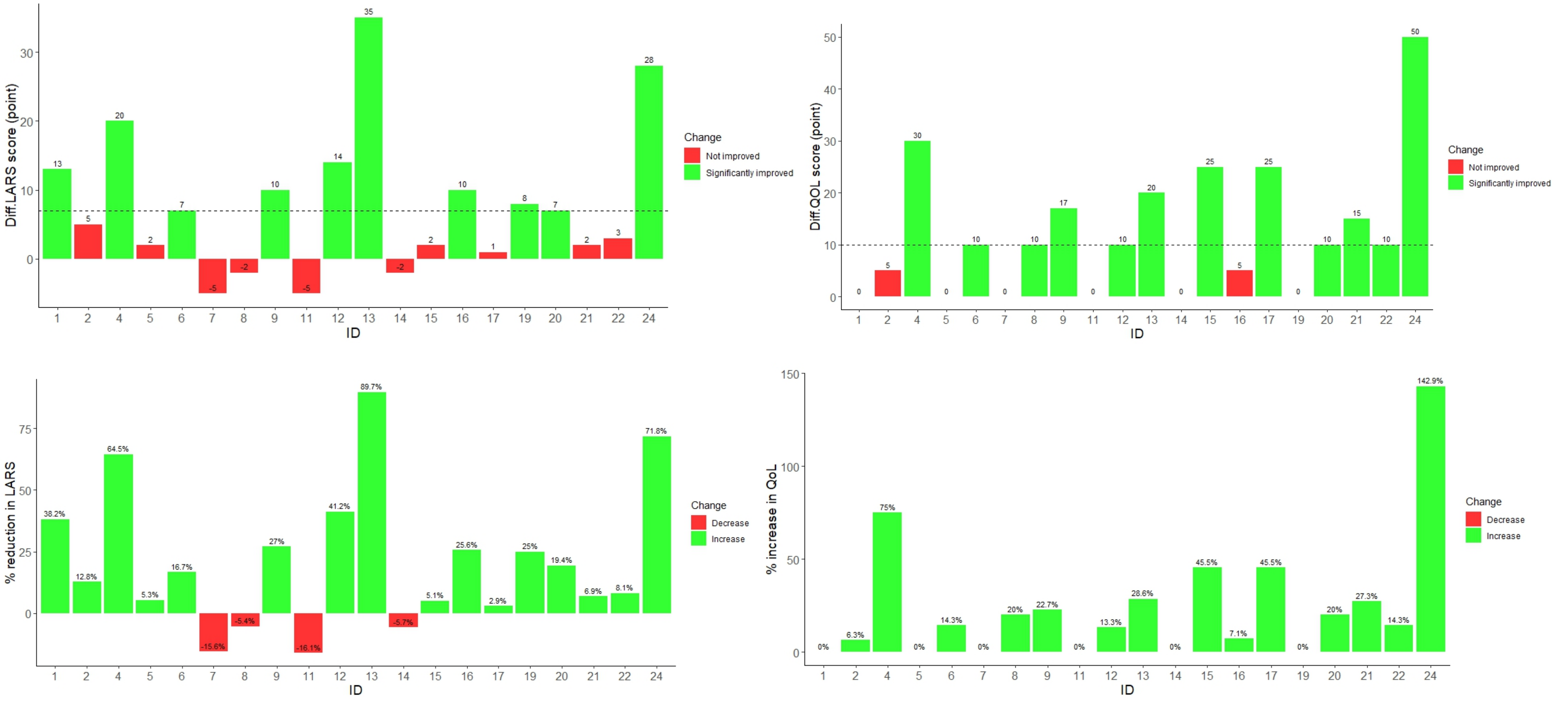

Figure 5. Supplementary. The individual changes in LARS scores (upper left) and EQ-VAS score (upper right) showing how many points each patient changed in scores during the treatment period. The individual change in % in LARS scores (lower left) and EQ-VAS score (lower right) showing 18 patients with a positive change in LARS score, while four patients had a negative change in LARS score. All 22 patients had an increased or unchanged QoL score. Green bars indicate individuals with a clinically meaningful change of score. Dashed lines represent the cut-off for clinically meaningful change.
